# Supplementary material for: CREB overexpression in dorsal CA1 ameliorates long-term memory deficits in aged rats
Source: eLife. 2017 Jan 4;6:e19358. doi: 10.7554/eLife.19358 (PMC5214885; doi:10.7554/eLife.19358)
Supplement: Supplementary file 1. — (a) Peak post-burst AHP amplitudes (mV) Aged CREB+ cells have smaller peak AHP’s than CREB- and GFP, but young cell types do not differ from each other. Data represent mean ± SEM. *p<0.05, **p<0.01 compared to aged CREB+. (b) Slow post-burst AHP amplitudes (mV) Aged CREB+ cells have smaller slow AHP’s than CREB- and GFP, but young cell types do not differ from each other. Data represent mean ± SEM. *p<0.05, **p<0.01 compared to aged CREB+. DOI: http://dx.doi.org/10.7554/eLife.19358.010 [file elife-19358-supp1.docx]

Supplementary File 1a. Peak post-burst AHP amplitudes (mV)

|  | CREB+ | CREB- | GFP |
| --- | --- | --- | --- |
| Young | -3.57 ± 0.70  (n = 9) | -4.74 ± 0.42  ( n = 17) | -3.64 ± 0.49  (n = 13) |
| Aged | -2.66 ± 0.39  (n = 7) | -5.20 ± 0.64*  (n = 9) | -5.35 ± 0.58**  (n =14) |

Aged CREB+ cells have smaller peak AHP’s than CREB- and GFP, but young cell types do not differ from each other. Data represent mean ± SEM. * p <0.05, ** p < 0.01 compared to aged CREB+.

Supplementary File 1b. Slow post-burst AHP amplitudes (mV)

|  | CREB+ | CREB- | GFP |
| --- | --- | --- | --- |
| Young | -1.18 ± 0.33  (n = 9) | -1.54 ± 0.17  (n = 17) | -1.09 ± 0.16  (n = 13) |
| Aged | -0.71 ± 0.14  (n = 7) | -1.83 ± 0.28*  (n = 9) | -1.88 ± 0.28**  (n = 14) |

Aged CREB+ cells have smaller slow AHP’s than CREB- and GFP, but young cell types do not differ from each other. Data represent mean ± SEM. * p <0.05, ** p < 0.01 compared to aged CREB+.
