# Supplementary material for: CREB overexpression in dorsal CA1 ameliorates long-term memory deficits in aged rats
Source: eLife. 2017 Jan 4;6:e19358. doi: 10.7554/eLife.19358 (PMC5214885; doi:10.7554/eLife.19358)
Supplement: Supplementary file 2. — (a) Resting Membrane Potential (mV) Resting membrane potential does not vary across different groups of cells. A two-way ANOVA revealed no significant effect of age (F1, 63 = 0.6078, n.s.) or of cell type (F2, 63 = 0.6122, n.s.). Data represent mean ± SEM. (b) Input Resistance (MΩ) Input resistance does not vary across different groups of cells. A two-way ANOVA revealed no significant effect of age (F1, 63 = 0.8259, n.s.) or of cell type (F2, 63 = 1.608, n.s.). Data represent mean ± SEM. DOI: http://dx.doi.org/10.7554/eLife.19358.011 [file elife-19358-supp2.docx]

Supplementary File 2a. Resting Membrane Potential (mV)

|  | CREB+ | CREB- | GFP |
| --- | --- | --- | --- |
| Young | -62.19 ± 2.28  (n = 9) | -62.04 ± 2.08  (n = 17) | -64.16 ± 2.39  (n = 13) |
| Aged | -58.23 ± 2.96  (n = 7) | -64.74 ± 1.50  (n = 9) | -60.20 ± 3.28  (n = 14) |

Resting membrane potential does not vary across different groups of cells. A two-way ANOVA revealed no significant effect of age (F_1, 63_ = 0.6078, n.s.) or of cell type (F_2, 63_ = 0.6122, n.s.). Data represent mean ± SEM.

Supplementary File 2b. Input Resistance (MΩ)

|  | CREB+ | CREB- | GFP |
| --- | --- | --- | --- |
| Young | 47.75 ± 5.18  (n = 9) | 53.51 ± 4.02  (n = 17) | 64.59 ± 10.95  (n = 13) |
| Aged | 51.68 ± 4.84  (n = 7) | 73.92 ± 11.40  (n = 9) | 57.40 ± 5.58  (n = 13) |

Input resistance does not vary across different groups of cells. A two-way ANOVA revealed no significant effect of age (F_1, 63_ = 0.8259, n.s.) or of cell type (F_2, 63_ = 1.608, n.s.). Data represent mean ± SEM.
